# Pathophysiology and emerging biomarkers of cardiovascular-renal-hepato-metabolic syndrome

**DOI:** 10.3389/fcvm.2025.1661563

**Published:** 2025-10-29

**Authors:** John Nzobokela, Lweendo Muchaili, Alick Mwambungu, Sepiso K. Masenga, Annet Kirabo

**Affiliations:** ^1^ Department of Pathology, Ndola Teaching Hospital, Ndola, Zambia; ^2^ HAND Research Group, School of Medicine and Health Sciences, Mulungushi University, Livingstone, Zambia; ^3^ Department of Cardiovascular Science and Metabolic Diseases, Livingstone Center for Prevention and Translational Science, Livingstone, Zambia; ^4^ Department of Biomedical Sciences, Ndola College of Biomedical Sciences, Ndola, Zambia; ^5^ Department of Molecular Physiology and Biophysics, Vanderbilt University Medical Center, Nashville, TN, United States; ^6^ Vanderbilt Institute for Global Health, Vanderbilt University Medical Center, Nashville, TN, United States; ^7^ Department of Medicine, Vanderbilt University Medical Center, Nashville, TN, United States; ^8^ Vanderbilt Center for Immunobiology, Vanderbilt University Medical Center, Nashville, TN, United States; ^9^ Vanderbilt Institute for Infection, Immunology and Inflammation, Vanderbilt University Medical Center, Nashville, TN, United States

**Keywords:** cardiovascular disease, chronic kidney disease, metabolic dysfunction-associated steatotic liver disease, metabolic disorders, inflammation, cardiovascular-renal-hepato-metabolic syndrome, metabolic syndrome

## Abstract

Cardiovascular-Renal-Hepatic-Metabolic (CRHM) syndrome characterizes a complex, interrelated disease framework that encompasses cardiovascular disease, chronic kidney disease (CKD), metabolic dysfunction-associated steatotic liver disease, and metabolic disorders such as obesity, type 2 diabetes mellitus, dyslipidemia, and hypertension. The syndrome extends the concept of cardiovascular-Kidney-Metabolic syndrome by incorporating the liver's pivotal role in systemic metabolic dysfunction. This syndrome progresses through a cycle of chronic inflammation, insulin resistance, oxidative stress, and endothelial dysfunction, driving multi-organ failure and increasing morbidity and mortality. Understanding the mechanistic keystones of this syndrome is critical for refining risk stratification and therapeutic interventions. Traditional inflammatory markers, such as C-reactive protein, interleukin-6, and tumor necrosis factor-alpha, have limitations in predicting long-term disease progression. Emerging biomarkers offer novel insights into systemic disease mechanisms and personalized medicine. Soluble urokinase plasminogen activator receptor has been identified as a stable and predictive marker of systemic inflammation, with strong associations with CKD, atherosclerosis, and coronary artery disease. Galectin-3 is a key regulator of fibrosis and inflammation across multiple organ systems, while Growth Differentiation Factor-15 has been implicated in mitochondrial dysfunction and cardiovascular aging. Furthermore, microRNAs such as miR-126 and miR-423-5p show promise as biomarkers for vascular integrity and heart failure progression, respectively. These biomarkers not only aid in early detection but also guide targeted interventions. Elevated levels of these markers support the use of sodium-glucose cotransporter 2 inhibitors for cardiorenal protection, and glucagon-like peptide-1 receptor agonists or dual glucose-dependent insulinotropic polypeptide and glucagon-like peptide 1 receptor agonists for metabolic and liver-related complications. Despite these advancements, the clinical integration of novel biomarkers remains limited. This review analyzes the pathophysiological mechanisms underlying CRHM syndrome and explores key biomarkers poised to enhance risk assessment and patient monitoring.

## 1 Introduction

In the early 2000s, studies linked metabolic syndrome as an assemblage of metabolic risk factors driving the development of cardiovascular disease (CVD) and the introduction of the term Cardiometabolic syndrome (CM) (1). Over the past 10 years, this concept has evolved dramatically as CM became a global pandemic, paving a path for further studies that recognized the interconnections with other conditions such as chronic kidney disease (CKD) and metabolic dysfunction-associated steatotic liver disease (MASLD). Cardiovascular Renal Hepatic Metabolic (CRHM) syndrome has emerged as a conceptual framework describing interconnected pathophysiological mechanisms (2). While not a recognized diagnosis, it provides valuable insights into shared disease processes and therapeutic strategies. These interconnected conditions intensify disease progression, elevating the risk of multi-organ dysfunction, morbidity, and mortality, thereby imposing a significant burden on healthcare systems (3).

**Figure 1:**
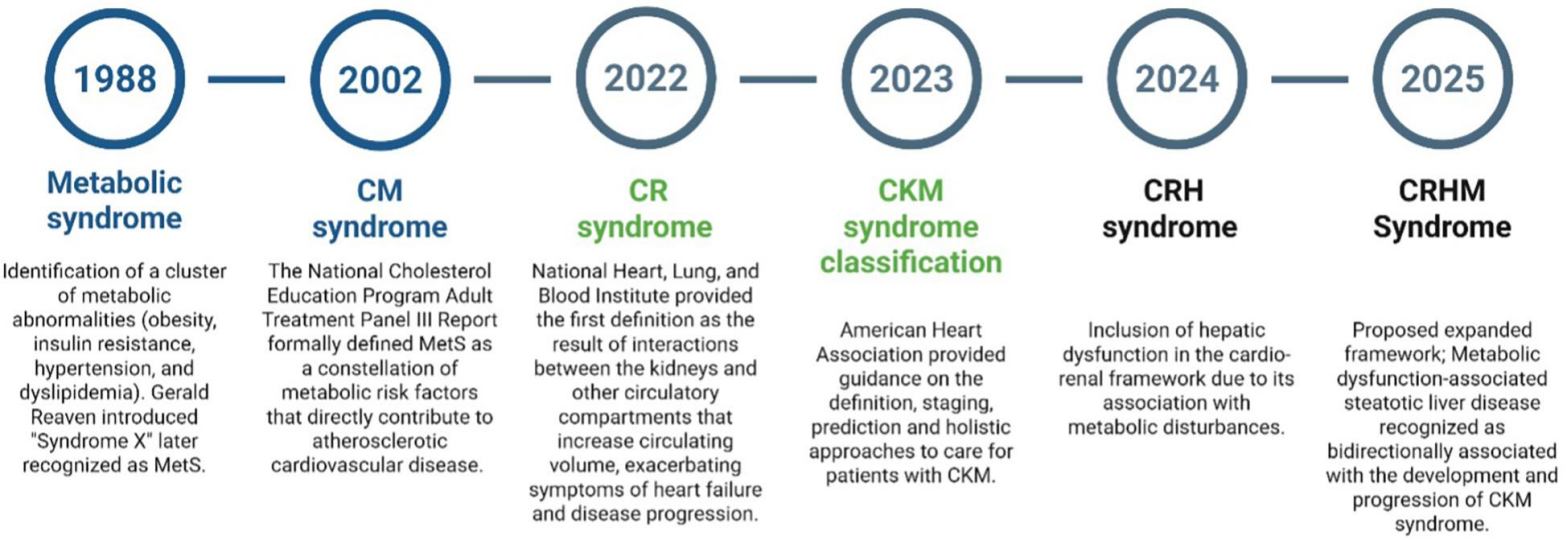
A chronological overview of the evolution of cardio-renal-hepatic-metabolic syndrome. MetS, metabolic syndrome; CM, cardiometabolic; CR, cardiorenal; CKM, cardiovascular kidney metabolic; CRH, cardiorenal hepatic; CRHM, cardiorenal, hepatic, and metabolic. Created using Biorender, licensed under Academic License.

Traditional inflammatory markers such as C-reactive protein (CRP), interleukin-6 (IL-6), and tumor necrosis factor-alpha (TNF-α) have been widely used for risk assessment; however, they lack specificity in predicting long-term disease outcomes (4). Soluble urokinase plasminogen activator receptor (suPAR) has emerged as a more stable and predictive biomarker of systemic chronic inflammation and, is associated with renal disease progression, cardiovascular risk, and metabolic disorders (5). Elevated suPAR levels have been shown to correlate with CKD, atherosclerosis, and coronary artery calcification, and genetic studies further linked suPAR to proinflammatory monocyte activation, implicating it in vascular dysfunction (6, 7). In addition to suPAR, galectin-3, a key regulator of fibrosis and inflammation, is strongly associated with cardiac remodeling, hepatic fibrosis, and kidney disease progression, with elevated levels linked to higher mortality in heart failure (HF) and liver fibrosis (8). Other emerging biomarkers, including growth differentiation factor-15 (GDF-15) and microRNAs (miRNAs), have been implicated in mitochondrial dysfunction, cardiovascular aging, and metabolic stress, with altered miRNA expression patterns correlating with atherosclerosis, insulin resistance, and cardiac dysfunction (9, 10).

Despite the increasing recognition of CRHM syndrome as a complex and interconnected disease entity, substantial gaps persist in its diagnosis, risk stratification, and long-term monitoring. The traditional compartmentalization of cardiology, nephrology, hepatology, and endocrinology has resulted in delayed diagnosis, suboptimal treatment coordination, and missed opportunities for early intervention. While emerging biomarkers offer valuable mechanistic insights into multi-organ dysfunction, their integration into routine clinical practice remains scarce.

Given the intricate interplay of multi-organ dysfunction in CRHM syndrome, advanced biomarkers provide new opportunities for refining risk stratification and therapeutic interventions. This review analyzes the pathophysiological mechanisms underlying CRHM syndrome, emphasizing the interaction between inflammation, metabolic dysregulation, and progressive organ dysfunction. Furthermore, we discuss the clinical utility of key biomarkers in diagnosing, monitoring, and managing CRHM syndrome, examining how these biomarkers can transform risk assessment, improve patient outcomes, and guide future therapeutic approaches.

## 2 The evolution of cardio-renal-hepatic-metabolic syndrome

Metabolic syndrome (MetS) is characterized by a cluster of interrelated risk factors for type 2 diabetes mellitus (T2DM) and CVD, including insulin resistance, obesity, hypertension, and dyslipidemia (11, 12). In 1988, Dr. Gerald Reaven introduced the term “Syndrome X” during his Banting Lecture, highlighting the role of insulin resistance in human disease; this concept was later recognized as MetS (Figure 1). MetS has since been identified as a significant risk factor for CVDs, substantially increasing the likelihood of heart disease, stroke, and type 2 diabetes (13, 14). The National Cholesterol Education Program (NCEP) Adult Treatment Panel III (ATP III) Report (2002) formally defined MetS as a constellation of metabolic risk factors that directly contribute to Atherosclerotic cardiovascular disease (ASCVD). ATP III emphasized the role of central obesity, insulin resistance, hypertension, dyslipidemia, and inflammation in driving cardiovascular risk (1). Later after 2002, Studies demonstrated that MetS and type 2 diabetes synergistically impact vascular thickness, leading to accelerated carotid intima-media thickness increase, a key marker of subclinical atherosclerosis (15, 16). The interaction of MetS components further amplifies vascular remodeling, increasing the risk of cardiovascular events.

In 2004, the National Heart, Lung, and Blood Institute provided the first definition of Cardio-Renal Syndrome (CRS) as “the result of interactions between the kidneys and other circulatory compartments that increase circulating volume, exacerbating symptoms of HF and disease progression.” (17). In 2008, the Acute Dialysis Quality Initiative consensus, led by Claudio Ronco and colleagues, categorized CRS into five subtypes based on primary organ dysfunction and the acute or chronic nature of the disease: Type 1, or acute CRS, involves the rapid onset of acute kidney injury (AKI) due to acute heart failure (AHF), while Type 2 refers to chronic heart failure progressively leading to CKD. In contrast, Type 3, known as acute reno-cardiac syndrome, describes acute kidney dysfunction triggering acute cardiac events, and Type 4, or chronic reno-cardiac syndrome, occurs when CKD contributes to chronic heart failure. Type 5, the secondary form, involves systemic conditions such as sepsis, amyloidosis, or cirrhosis that concurrently impair both renal and cardiac function (18).

Building upon the foundation of MetS, in 2023, the American Heart Association (AHA) introduced the cardiovascular-kidney-metabolic (CKM) Syndrome, which was defined as: A systemic disorder characterized by pathophysiological interactions among metabolic risk factors, CKD, and the cardiovascular system, leading to multiorgan dysfunction and high rates of adverse cardiovascular outcomes. The AHA provided guidance on definition, staging, prediction, and holistic approaches to care for patients with CKM syndrome. By 2024, research expanded the cardio-renal framework to include hepatic dysfunction, recognizing its role in metabolic disturbances disturbances (19). Traditionally, Hepato-Renal Syndrome was considered kidney failure secondary to liver dysfunction. However, emerging evidence suggests that subtle cardiac abnormalities often precede and predict the development of kidney disease in patients with liver cirrhosis (20). This evolving understanding led to the conceptualization of Cardio-Renal-Hepato (CRH) syndrome.

In February 2025, Theodorakis and Nikolaou proposed an expanded framework transitioning from CKM to CRHM syndrome (2). While the CKM model addressed the interplay between cardiovascular, renal, and metabolic systems, it did not fully capture the role of the liver. Given the increasing recognition of MASLD and its progression to metabolic dysfunction-associated steatohepatitis (MASH) and cirrhosis, an integrated model was necessary. The proposed CRHM Syndrome is defined as: “A systemic disorder that leads to parallel multiorgan dysfunction driven by shared pathophysiological mechanisms, including metabolic inflammation (meta-inflammation) and dysregulation, particularly insulin resistance” (2, 21). Building upon this framework, we propose a more integrated diagnostic approach, utilizing emerging biomarkers, advanced risk stratification, and clinical insights to optimize patient outcomes and drive precision medicine in CRHM syndrome.

## 3 Pathophysiology of cardio-renal-hepatic-metabolic syndrome

**Figure 2:**
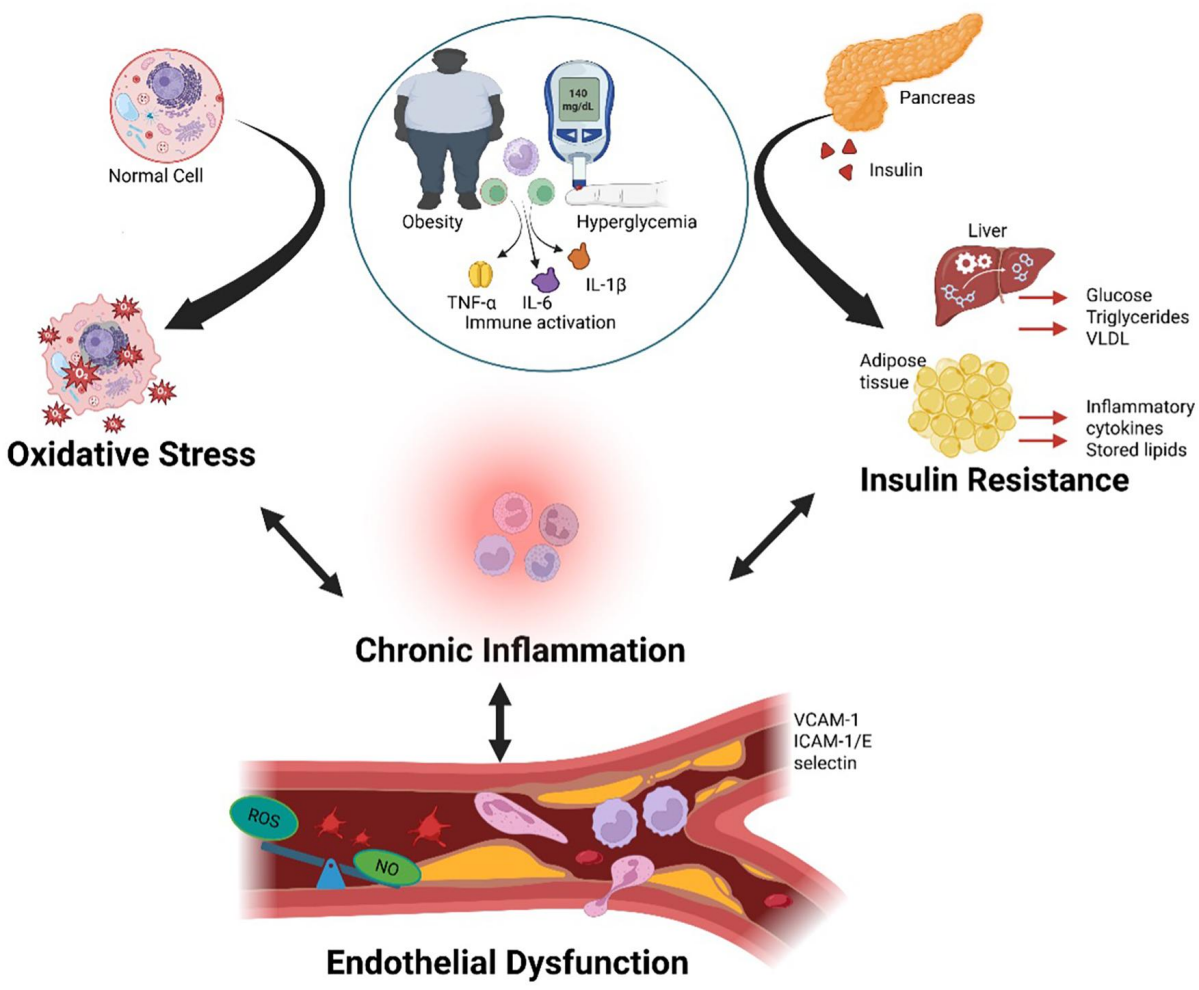
Obesity and hyperglycemia promote systemic inflammation and metabolic organ dysfunction. Obesity and hyperglycemia synergistically activate the immune system by increasing circulating pro-inflammatory cytokines such as TNF-α, IL-6, and IL-1β, leading to chronic systemic inflammation. This inflammatory state alters immune cell profiles, promotes ROS production, and NO signaling within blood vessels, resulting in vascular dysfunction. Persistent inflammation contributes to dysfunction in key metabolic organs including the pancreas, liver, and adipose tissue, thereby reinforcing the cycle of metabolic dysregulation and immune activation. TNF-α, tumor necrosis factor-alpha; IL-6, interleukin-6; IL-1β, interleukin-1 beta; ROS, reactive oxygen species; NO, nitric oxide. Created using Biorender, licensed under Academic License.

The pathophysiology of CRHM syndrome is driven by a multifaceted interaction of unified mechanisms, creating a self-perpetuating cycle of multi-organ dysfunction. At its core, chronic inflammation acts as the foundation, initiating tissue damage through the release of pro-inflammatory cytokines, immune activation, and fibrotic changes (2, 22). Insulin resistance fuels this process, worsening metabolic dysfunction, hyperglycemia, and lipid dysregulation, which further strain the cardiovascular, renal, hepatic, and metabolic systems (22, 23). As the condition progresses, oxidative stress amplifies the damage, increasing cellular injury, mitochondrial dysfunction, and reactive oxygen species (ROS) production, worsening organ failure (24). Finally, endothelial dysfunction closes the loop, impairing vascular integrity, increasing arterial stiffness, and perpetuating ischemic injury, which leads to additional CVD, CKD, hepatic fibrosis, and metabolic dysregulation (25). Together, these mechanisms create a cycle that drives CRHM syndrome, making it a progressive and challenging condition that requires a multi-targeted therapeutic approach Figure 2.

### 3.1 Inflammation: the foundation of cardio-renal-hepatic-metabolic syndrome

Obesity-induced adipose tissue dysfunction initiates a chronic low-grade inflammatory state, or “meta-inflammation,” which plays a pivotal role in the development of cardio-renal-metabolic diseases. As adipose tissue expands, hypoxia and cellular stress trigger adipocyte death and recruit pro-inflammatory M1 macrophages, replacing anti-inflammatory M2 macrophages (26, 27). This phenotypic shift exacerbates inflammation and disrupts metabolic homeostasis. M1 macrophages secrete pro-inflammatory cytokines such as TNF-α and IL-6, which impair insulin receptor signaling by activating serine kinases like c-Jun N-terminal kinase (JNK) and inhibitor of kappa B kinase-beta (IKK-β). These kinases phosphorylate insulin receptor substrates (IRS), reducing glucose transporter type 4 (GLUT4) -mediated glucose uptake and contributing to hyperglycemia and systemic insulin resistance (28, 29).

**Figure 3:**
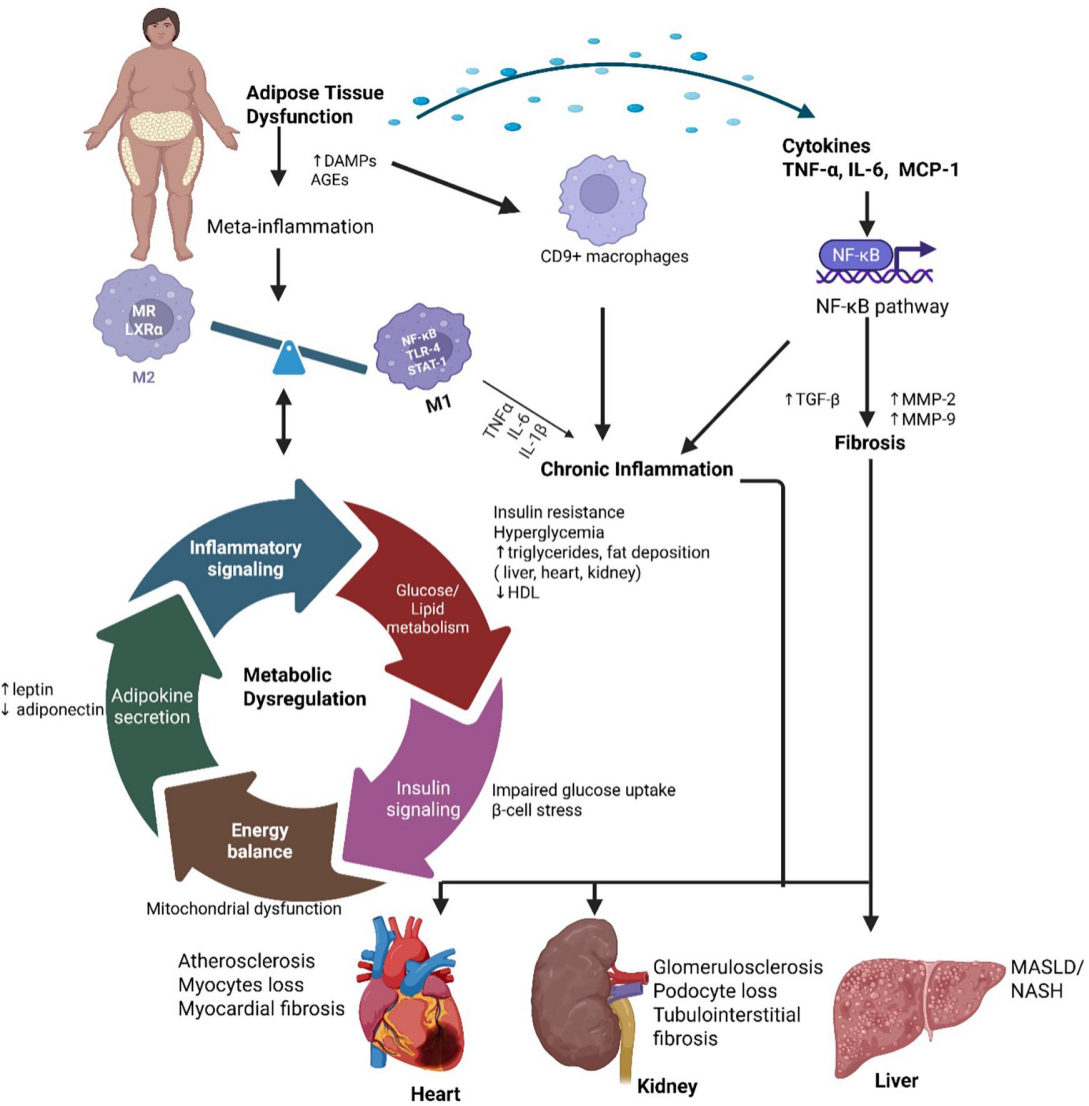
Meta-inflammation and metabolic dysregulation in cardio-renal-metabolic disease. Obesity-induced adipose tissue expansion leads to hypoxia, cellular stress, and adipocyte death, promoting the recruitment of pro-inflammatory M1 macrophages via activation of NF-κB, TLR4, and STAT1 signaling, while reducing anti-inflammatory M2 macrophages characterized by MR and LXRα expression. This immune imbalance drives chronic low-grade inflammation (“meta-inflammation”) and disrupts key metabolic processes, including glucose/lipid metabolism, insulin signaling, adipokine secretion, and energy balance. These alterations contribute to systemic metabolic dysregulation and end-organ damage in the heart, kidneys, and liver. TNF-α, tumor necrosis factor-alpha; IL-6, interleukin-6; MCP-1, monocyte chemoattractant protein-1; NF-κB, nuclear factor-kappa B; TLR4, toll-like receptor 4; STAT1, signal transducer and activator of transcription 1; MR, mannose receptor; LXRα, liver X receptor alpha; M1/M2, classically/alternatively activated macrophages. Created using Biorender, licensed under Academic License.

The nuclear factor-kappa B **(**NF-κB) pathway plays a crucial role in obesity-associated inflammation. Under normal conditions, NF-κB proteins are sequestered in the cytoplasm by inhibitors of κB (IκBs). However, activation of the IKK kinase complex results in IκB degradation, facilitating NF-κB nuclear translocation and upregulation of pro-inflammatory genes, including Interferon gamma (IFN-γ), transforming growth factor beta (TGF-β), interleukin (IL)-1β, monocyte chemoattractant protein 1 (MCP-1), IL-6 and TNF-α. This cascade amplifies inflammation and fibrosis. Additionally, hypertrophic adipocytes release TNF-α, IL-6, and MCP-1, reinforcing the inflammatory state and perpetuating insulin resistance Figure 3 (30, 31).

Recent single-cell transcriptomic studies have identified distinct macrophage populations such CD9 + macrophages in obese adipose tissue that display both inflammatory and pro-fibrotic phenotypes (32, 33). These adipose-derived cytokines enter the circulation and contribute to systemic inflammation, cardiac remodeling, renal fibrosis, and hepatic steatosis (34, 35). The pathogenic loop between adipose inflammation and organ damage underlies insulin resistance and progression of obesity-associated chronic diseases. Macrophages, T-cells, and neutrophils infiltrate inflamed during chronic low-grade inflammation, contributing to the progression of cardio-renal-hepatic-metabolic syndrome. Upon infiltration, these immune cells release ROS, matrix metalloproteinases (MMPs), and fibrogenic cytokines such as TGF-β, which collectively exacerbate tissue damage and fibrosis. ROS, primarily generated by nicotinamide adenine dinucleotide phosphate (NADPH) oxidase in activated immune cells, induce oxidative stress, lipid peroxidation, protein modification, and mitochondrial dysfunction, ultimately triggering apoptosis and compromising cellular function in metabolic and cardiovascular tissues (36, 37).

MMPs, such as MMP-2 and MMP-9, are activated by pro-inflammatory cytokines and degrade components of the extracellular matrix, thereby disrupting tissue integrity and enhancing inflammation through activation of NF-κB and activating protein-1 (AP-1) signaling pathways (38–40). TGF-β also acts as a master regulator of fibrosis by stimulating fibroblast proliferation and differentiation into myofibroblasts via the SMAD2/3 signaling cascade. This leads to excessive collagen production and extracellular matrix (ECM) accumulation (41). The prolonged activation of these inflammatory and fibrotic pathways promotes progressive tissue remodeling, multi-organ dysfunction, and the systemic complications characteristic of CRHM syndrome.

### 3.2 Insulin resistance: the fuel for cardio-renal-hepatic-metabolic syndrome

Insulin resistance is a crucial driver of the syndrome, acting as both a consequence and amplifier of chronic inflammation. In normal conditions, insulin regulates glucose metabolism, lipid balance, and endothelial function. However, in insulin-resistant states, cells in the liver, muscles, and adipose tissue fail to respond efficiently to insulin, leading to hyperglycemia, dyslipidemia, and metabolic stress. This dysfunction fuels multi-organ damage and perpetuates oxidative stress, inflammation, and endothelial dysfunction (29, 42).

Meta-inflammation in obesity disrupts insulin signaling through alterations in adipokine secretion and excessive free fatty acid (FFA) release, forming a pro-inflammatory microenvironment that impairs insulin sensitivity and reduces glucose uptake in peripheral tissues (43). Usually, adipokines such as adiponectin improve insulin sensitivity by activating the AMP-activated protein kinase (AMPK) pathway, promoting fatty acid oxidation and glucose uptake. However, chronic inflammation suppresses adiponectin levels while increasing leptin, resistin, and TNF-α production, which interfere with insulin receptor function (44). Leptin overexpression activates Janus kinase-signal transducer and activator of transcription (JAK-STAT) signaling, leading to the induction of suppressor of cytokine signaling 3 (SOCS-3), which inhibits IRS-1 and IRS-2, impairing downstream phosphoinositide 3-kinase-protein kinase B (PI3K-Akt) signaling essential for glucose uptake. Resistin, through TLR4-NF-κB activation, upregulates inflammatory cytokines, sustaining chronic immune responses that exacerbate insulin resistance (45–47).

**Figure 4:**
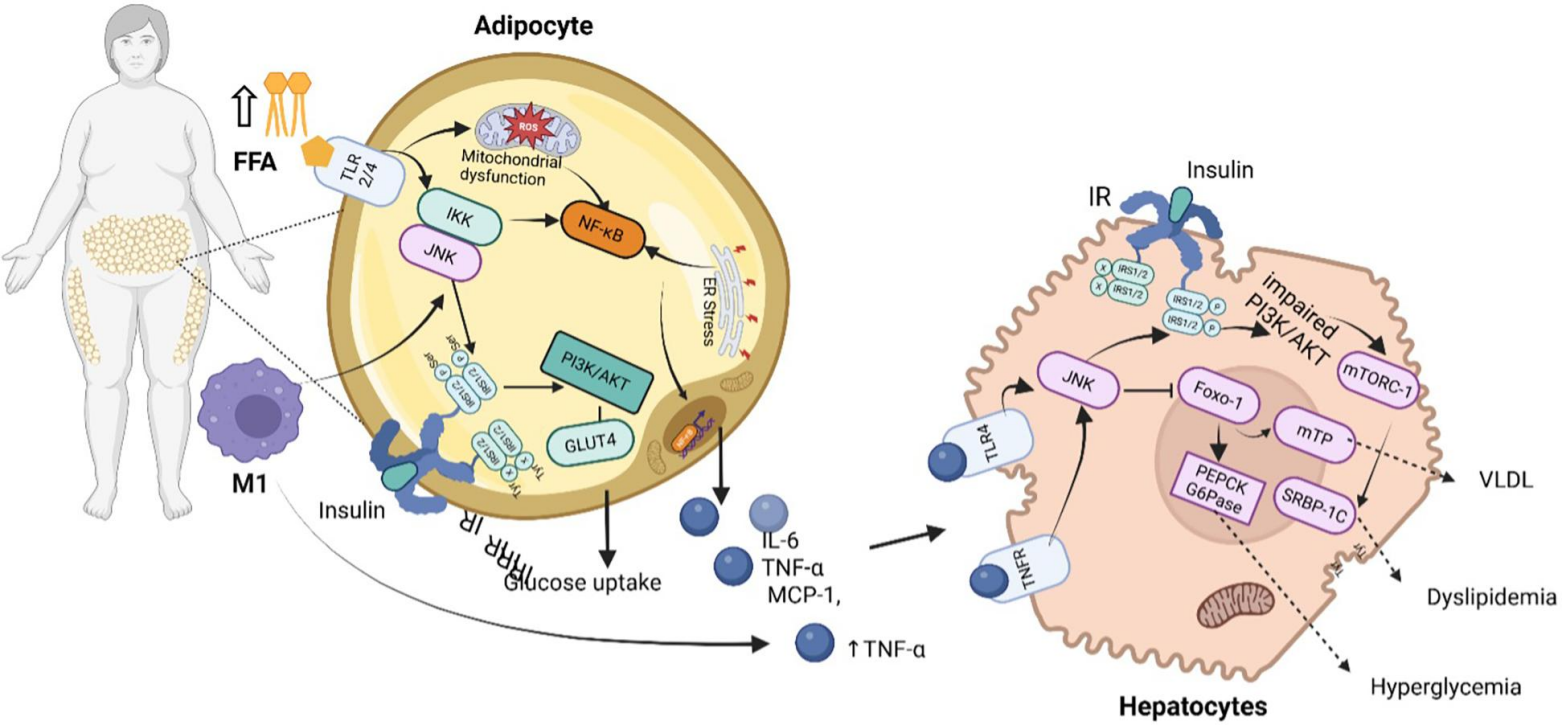
Inflammatory and metabolic dysregulation pathways driving CRHM syndrome. In obese individuals, increased FFAs and activation of immune cells (M1 macrophages) stimulate inflammatory pathways such as NF-κB and JNK in adipocytes, resulting to mitochondrial dysfunction, ER stress, and reduced glucose uptake due to impaired PI3K/AKT signaling and decreased GLUT4 activity. These adipocytes release pro-inflammatory cytokines (IL-6, TNF-α, MCP-1), which further impair insulin signaling in hepatocytes. In the liver, this results in activation of gluconeogenic enzymes (PEPCK, G6Pase) and lipid metabolism regulators (SREBP-1C, mTORC-1), promoting increased VLDL production, hyperglycemia, and dyslipidemia. IR indicate insulin receptor; IRS1/2, insulin receptor substrate 1/2; TNFR, tumor necrosis factor receptor; TNF-α, tumor necrosis factor-alpha; TLR4/2, toll-like receptor 4/2; FFA, free fatty acids; IKK, inhibitor of nuclear factor kappa-B Kinase; PI3K/AKT, phosphoinositide 3-kinase/protein kinase B pathway; NF-κB, nuclear factor kappa-light-chain-enhancer of activated B Cells; ER, endoplasmic reticulum; ROS, reactive oxygen species; IL-6, interleukin-6; IFN-γ, interferon-gamma; TGF-β, transforming growth factor beta; MCP-1, monocyte chemoattractant protein-1; IL-6, interleukin-6; VLDL, very low-density lipoprotein. Created using Biorender, licensed under Academic License.

Beyond adipokine dysregulation, adipose tissue dysfunction leads to an increased release of FFAs into the bloodstream. Elevated FFAs exacerbate insulin resistance by promoting lipid accumulation in non-adipose tissues like skeletal muscle and the liver, where they interfere with insulin receptor signaling. FFAs also activate inflammatory pathways, including NF-κB and JNK, and further activate protein kinase c beta II (PKC-βII) and protein kinase c delta (PKC-δ) in human muscle, which disrupts insulin signaling by impairing IRS function, further propagating hyperglycemia and metabolic dysfunction (48, 49). The cumulative effect of these disruptions in both adipokine signaling and lipid metabolism impairs insulin sensitivity, contributing to the progression of T2DM (50). Insulin resistance disrupts hepatic glucose regulation, resulting in excessive glucose production, worsening hyperglycemia, and amplifying metabolic dysfunction. In insulin-sensitive states, insulin suppresses gluconeogenesis and promotes glycogen synthesis via PI3K-Akt activation, which inhibits forkhead box O1 (FOXO1), a key transcription factor regulating gluconeogenic enzymes (51). However, in insulin resistance, reduced IRS-1/PI3K-Akt signaling fails to inhibit FOXO1, leading to excessive expression of phosphoenolpyruvate carboxykinase (PEPCK) and glucose-6-phosphatase (G6Pase), which drive hepatic glucose overproduction (52). Concurrently, liver insulin resistance promotes *de novo* lipogenesis (*de novo* lipogenesis through sterol regulatory element-binding protein 1c (SREBP-1c) activation, increasing the synthesis of triglycerides and very-low-density lipoproteins (VLDL), contributing to MASLD and systemic dyslipidemia Figure 4 (50, 52, 53).

CRHM Syndrome-associated dyslipidemia is marked by elevated triglycerides, reduced high-density lipoprotein (HDL), and increased small-dense low-density lipoprotein (LDL), compounding cardiovascular risk. Insulin resistance impairs lipoprotein lipase activity, reducing peripheral lipid clearance, while hepatic overproduction of VLDL leads to an accumulation of triglyceride-rich lipoproteins in circulation (54). Impaired lipid storage in adipose tissue leads to excess circulating FFAs, which deposit in non-adipose organs such as the liver, heart, and kidneys, driving lipotoxicity and cellular dysfunction. In hepatocytes, excessive FFAs activate PKCε, which inhibits insulin receptor phosphorylation, worsening insulin resistance and mitochondrial dysfunction (55). In cardiomyocytes, lipid overload leads to excessive β-oxidation, generating ROS that induce mitochondrial stress, cardiomyocyte apoptosis, and fibrosis, contributing to diabetic cardiomyopathy (56). Similarly, in the kidneys, lipid accumulation disrupts podocyte function and induces inflammation through toll-like receptor 4 (TLR4)-NF-κB activation, exacerbating glomerular damage and chronic kidney disease (57).

### 3.3 Oxidative stress: the reinforcer of cardio renal hepatic metabolic syndrome

**Figure 5:**
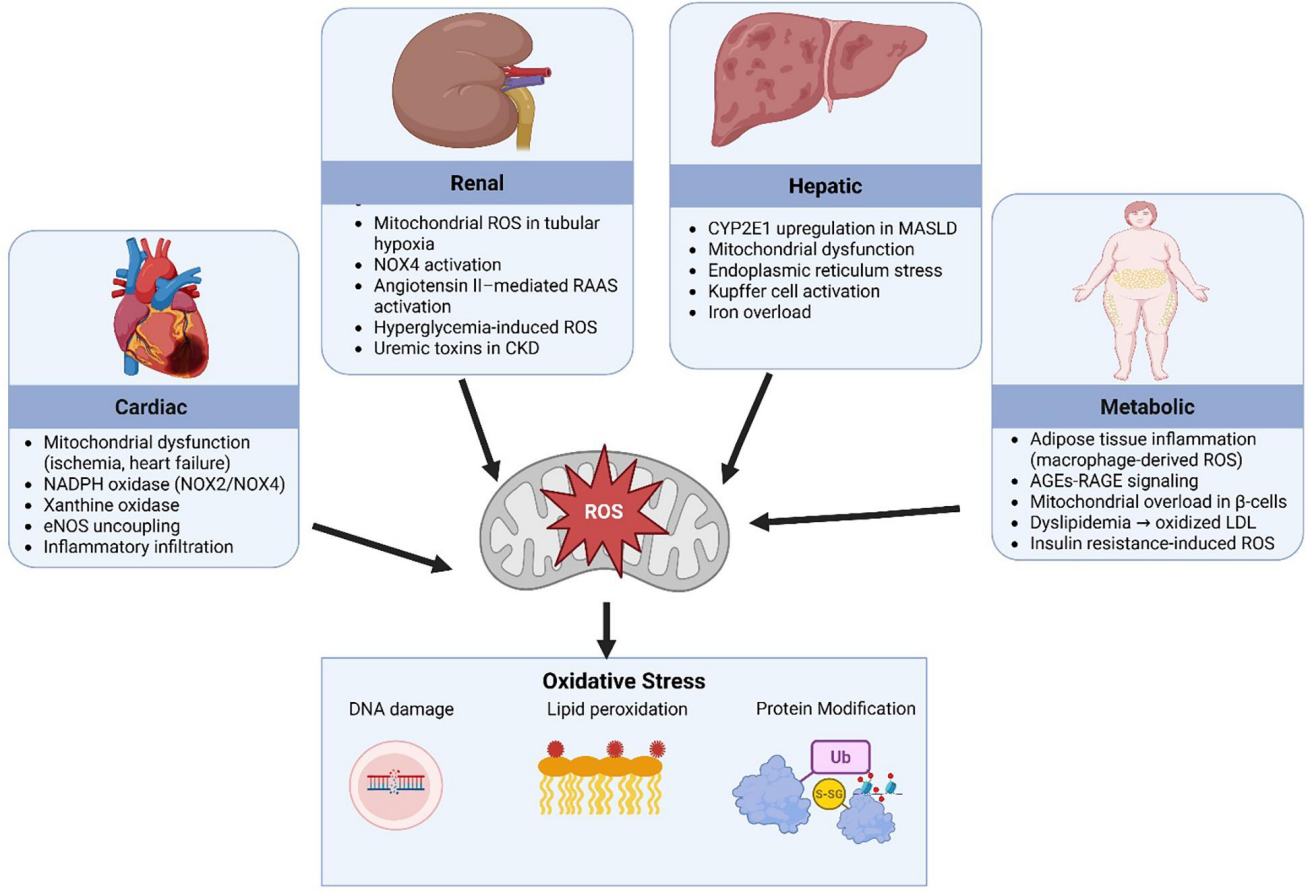
Sources of reactive oxygen species. SOD2, superoxide dismutase 2; ROS, reactive oxygen species; ER, endoplasmic reticulum; NADPH, nicotinamide adenine dinucleotide phosphate; H₂O₂, hydrogen peroxide; NOX2/NOX4, NADPH oxidase isoforms 2 and 4; RAAS, renin angiotensin aldosterone system; eNOS, endothelial nitric oxide synthase; CKD, chronic kidney disease; MASLD, metabolic dysfunction-associated steatotic liver disease; AGEs, advanced glycation end-products; RAGE, receptor for advanced glycation end-products; LDL, low-density lipoprotein. Created using Biorender, licensed under Academic License.

Oxidative stress plays an important role in the pathogenesis of CRHM syndrome as it acts as both a consequence and a reinforcer of the disease's progression. Under physiological conditions, ROS are neutralized by endogenous antioxidant systems which include superoxide dismutase (SOD), catalase, and glutathione peroxidase (58). However, in CRHM syndrome, the excessive production of ROS overwhelms these defenses with consequent oxidative damage to lipids, proteins, and DNA, thereby perpetuating organ dysfunction (59). In the heart, ROS generation arises primarily from mitochondrial dysfunction during ischemia and heart failure, with additional contributions from NADPH oxidase (NOX2/NOX4) and xanthine oxidase pathways, promoting cardiomyocyte injury and fibrosis (60, 61). The kidney contributes through hypoxia-induced mitochondrial ROS, NOX4 activation, and angiotensin II-stimulated renin angiotensin aldosterone system (RAAS) pathways, which worsen tubular damage and accelerate CKD progression (62, 63). Hepatic ROS production is intensified by cytochrome P450 2E1 (CYP2E1) upregulation in MASLD, Kupffer cell activation, and endoplasmic reticulum stress, all of which potentiate hepatocyte injury and inflammation (64, 65). In metabolic tissues, especially adipose tissue, macrophage-derived ROS, advanced glycation end-products (AGEs), which are proteins or lipids that undergo glycation when exposed to sugars, leading to the formation of potentially harmful end-products, interact with the receptor for advanced glycation end-products (RAGE), and insulin resistance-induced mitochondrial overload in β-cells drive systemic oxidative stress and dyslipidemia, contributing to endothelial dysfunction and atherosclerosis (66–69). The cumulative impact of these sources results in widespread oxidative damage, including DNA fragmentation, lipid peroxidation, and protein modifications, forming a vicious cycle of organ crosstalk and progressive dysfunction Figure 5.

Multiple interrelated stimuli such hyperglycemia, hyperlipidemia, inflammatory cytokines, and angiotensin II increase the production of ROS. This occurs through several mechanisms, including mitochondrial dysfunction, the formation of AGEs, and dysregulated cellular metabolism (70). The kidneys and liver, owing to their high metabolic activity, are particularly vulnerable to ROS-induced injury. In metabolic tissues, particularly adipose and skeletal muscle, ROS impair insulin signaling by altering adipokine profiles (decreased adiponectin and increased leptin/resistin) and by inhibiting key signaling molecules like IRS-1 and GLUT4, resulting in systemic insulin resistance. In the pancreas, β-cells, which have inherently low antioxidant defenses, undergo oxidative damage that impairs insulin secretion and promotes apoptosis. This combination of insulin resistance and β-cell failure further amplifies hyperglycemia, creating a vicious cycle of metabolic dysfunction and ROS production that fuels widespread cellular injury and inflammation across multiple organ systems. In the kidney, elevated ROS levels contribute to pathological changes such as glomerulosclerosis, tubular atrophy, and interstitial fibrosis, primarily through the activation of pro-inflammatory and pro-fibrotic signaling pathways, such as NF-κB and TGF-β (71). ROS directly oxidize and IKK complex, leading to phosphorylation and degradation of IκBα, which releases NF-κB for nuclear translocation. Once in the nucleus, NF-κB upregulates pro-inflammatory cytokines including TNF-α, IL-6, and IL-1β, creating a sustained inflammatory microenvironment (72). Simultaneously, ROS stimulate the Smad2/3 pathway by activating latent TGF-β through oxidative modification of its latency-associated peptide (LAP). Phosphorylated Smad2/3 complexes then translocate to the nucleus, where they induce transcription of fibrogenic genes, including collagen I/III, fibronectin, and α-smooth muscle actin (α-SMA). This oxidative stress-induced crosstalk between NF-κB-mediated inflammation and TGF-β/Smad-driven fibrogenesis creates a self-amplifying loop that perpetuates glomerulosclerosis, tubular atrophy, and interstitial fibrosis in CRHM syndrome (73).

Similarly, hepatic oxidative stress accelerates lipid peroxidation, mitochondrial dysfunction, and hepatocyte apoptosis, exacerbating MASLD and cirrhosis which are key components of CRHM syndrome. These lipid peroxidation products form protein adducts that will impair mitochondrial electron transport chain function, leading to further ROS generation and thereby creating a self-perpetuating cycle of oxidative damage (74). The most reactive and toxic byproducts of lipid peroxidation are 4-hydroxynonenal (4-HNE) and malondialdehyde (MDA), which covalently modify cysteine, histidine, and lysine residues on critical mitochondrial proteins, disrupting complexes I and III of the respiratory chain (75, 76). This worsens electron leakage and ROS production, triggers apoptosis and inflammation, and accelerates hepatocyte dysfunction and fibrosis in CRHM syndrome. Their persistent accumulation establishes a pathological feedback loop that will drive MASLD progression from steatosis to cirrhosis (77). In the cardiovascular system, ROS diminish NO bioavailability, promoting endothelial dysfunction, vascular stiffness, and atherosclerosis. ROS also activate pro-fibrotic and hypertrophic pathways (TGF-β, MAPKs), contributing to cardiac remodeling and heart failure (78, 79).

Recent studies have highlighted the detrimental effects of ROS on protein structures and functions, through oxidative modifications such as carbonylation and disulfide bond formation (80). These modifications can lead to protein misfolding, loss of enzymatic activity, impaired receptor function, and enhanced protein degradation (81, 82). In metabolic tissues, such as adipose tissue, increased ROS levels have been associated with the carbonylation of key mitochondrial proteins, including the phosphate carrier protein and NADH dehydrogenase (83). This carbonylation impairs mitochondrial respiration, increases superoxide production, and disrupts insulin signaling pathways, contributing to metabolic dysregulation and the progression of metabolic syndrome (84). Furthermore, protein carbonylation is considered an irreversible and irreparable modification, making it a significant marker of oxidative stress and protein damage in various diseases, including diabetes and cardiovascular disorders (84). In addition, Hyperglycemia drives mitochondrial overproduction of ROS, disrupting insulin signaling pathways, worsening metabolic dysfunction (70). In CRHMS, chronic hyperglycemia and excessive FFA flux drive mitochondrial dysfunction, leading to electron transport chain (ETC) overload and excessive superoxide (O₂^−^) production (85). This oxidative stress inactivates IRS-1 via serine phosphorylation, impairing insulin-dependent GLUT4 translocation (86). On the other hand, ROS activate NF-*κ*B and NLRP3 inflammasomes, upregulating IL-1β, IL-18, and TNF-α, which sustain systemic inflammation (87). In the liver, excess FFA induces steatosis and contributes to CYP2E1-mediated oxidative damage (88). In the kidney, NOX4 activation and mitochondrial ROS production further worsen glomerulosclerosis (89, 90).

Recent evidence highlights the crucial role of DNA damage in hindering cellular repair and regeneration in cardiovascular, renal, and metabolic tissues, thereby contributing to tissue dysfunction. In the cardiovascular system, DNA damage in cardiomyocytes has been strongly linked to the progression of heart failure (91). Single-cell RNA sequencing and myocardial biopsy analyses in patients with dilated cardiomyopathy revealed that those who failed to achieve left ventricular reverse remodeling exhibited significantly higher levels of DNA damage markers poly(ADP-ribose) and γ-H2A.X compared to those with LVRR, with %PAR at 16.3% vs. 3.7% and %γ-H2A.X at 11.7% vs. 3.5% (*p* < 0.0001) (91, 92). These markers independently predicted poor prognosis and adverse cardiovascular events, confirming DNA damage as a key prognostic indicator in dilated cardiomyopathy. In renal tissues, DNA damage in proximal tubular epithelial cells has been associated with systemic metabolic dysfunction. Research demonstrated that DNA double-strand breaks in proximal tubular epithelial cells led to weight loss, reduced fat mass, impaired glucose tolerance, mitochondrial dysfunction, and increased inflammation in adipose tissues, suggesting that DNA damage in kidney cells can have far-reaching effects on overall metabolic health (93). Furthermore, oxidative DNA damage, characterized by the formation of 8-oxoguanine, has been implicated in renal tubular epithelial cell injury. If not adequately repaired, such damage can lead to cell death, DNA mutations, and genomic instability, exacerbating kidney dysfunction (94).

### 3.4 Endothelial dysfunction: closing the loop in cardio renal hepatic metabolic syndrome

The proposed mechanisms linking chronic inflammation to endothelial dysfunction in CRHM syndrome include the suppression of endothelial nitric oxide synthase (eNOS) activity, increased expression of adhesion molecules, and cytokine-induced phenotypic switching of endothelial cells. TNF-α, IL-6, and CRP mediate the inflammatory shift of endothelial cells from a quiescent to a pro-inflammatory and pro-thrombotic phenotype (95). These cytokines inhibit eNOS expression and activity, resulting in reduced nitric oxide (NO) bioavailability, a key vasodilator and anti-inflammatory molecule. The subsequent NO deficiency impairs flow-mediated dilation and promotes vasoconstriction, increasing vascular tone and contributing to hypertension.

In states of insulin resistance, hepatic steatosis, and renal impairment, mitochondrial electron transport chain dysfunction drives the excessive production of ROS, which results in redox imbalance and metabolic stress (96, 97). Concurrently, activation of NOX2 and NOX4, increases O₂^−^ production that reacts rapidly with nitric oxide (NO) to generate peroxynitrite (ONOO^−^), a highly reactive nitrogen species that impairs NO bioavailability and exerts cytotoxic and pro-inflammatory effects on vascular cells (97–99). Beyond its damaging effects, ROS also modulates gene expression by stabilizing hypoxia-inducible factor-1*α* (HIF-1*α*) and promote the transcription of vascular endothelial growth factor (VEGF), thereby initiating angiogenesis under conditions of hypoxia and inflammation (100, 101). Therefore, there is a dual effect of ROS on vascular homeostasis contributing to endothelial dysfunction and vascular inflammation, ROS is also promoting pathological angiogenesis and neovascularisation (102).

Hyperinsulinemia and chronic inflammation, common in insulin resistance and type 2 diabetes, promote the overexpression and secretion of endothelin-1 (ET-1), a potent endothelium-derived vasoconstrictor. This upregulation occurs through the activation of PKC and MAPK signaling pathways, which also suppress eNOS activity, reducing NO bioavailability and exacerbating endothelial dysfunction (103, 104). Elevated plasma ET-1 levels are consistently associated with impaired vasodilation, increased systemic vascular resistance, and glomerular hypertension. Recent evidence show that ET-1 levels correlate positively with blood pressure and albuminuria in individuals with insulin resistance and early-stage chronic kidney disease (104). Furthermore, blockade of ET-1 receptors, mainly ETA, using antagonists such as atrasentan has demonstrated efficacy in reducing blood pressure and proteinuria, supporting the role of ET-1 in vascular and renal injury in CRHM syndrome (105).

**Figure 6:**
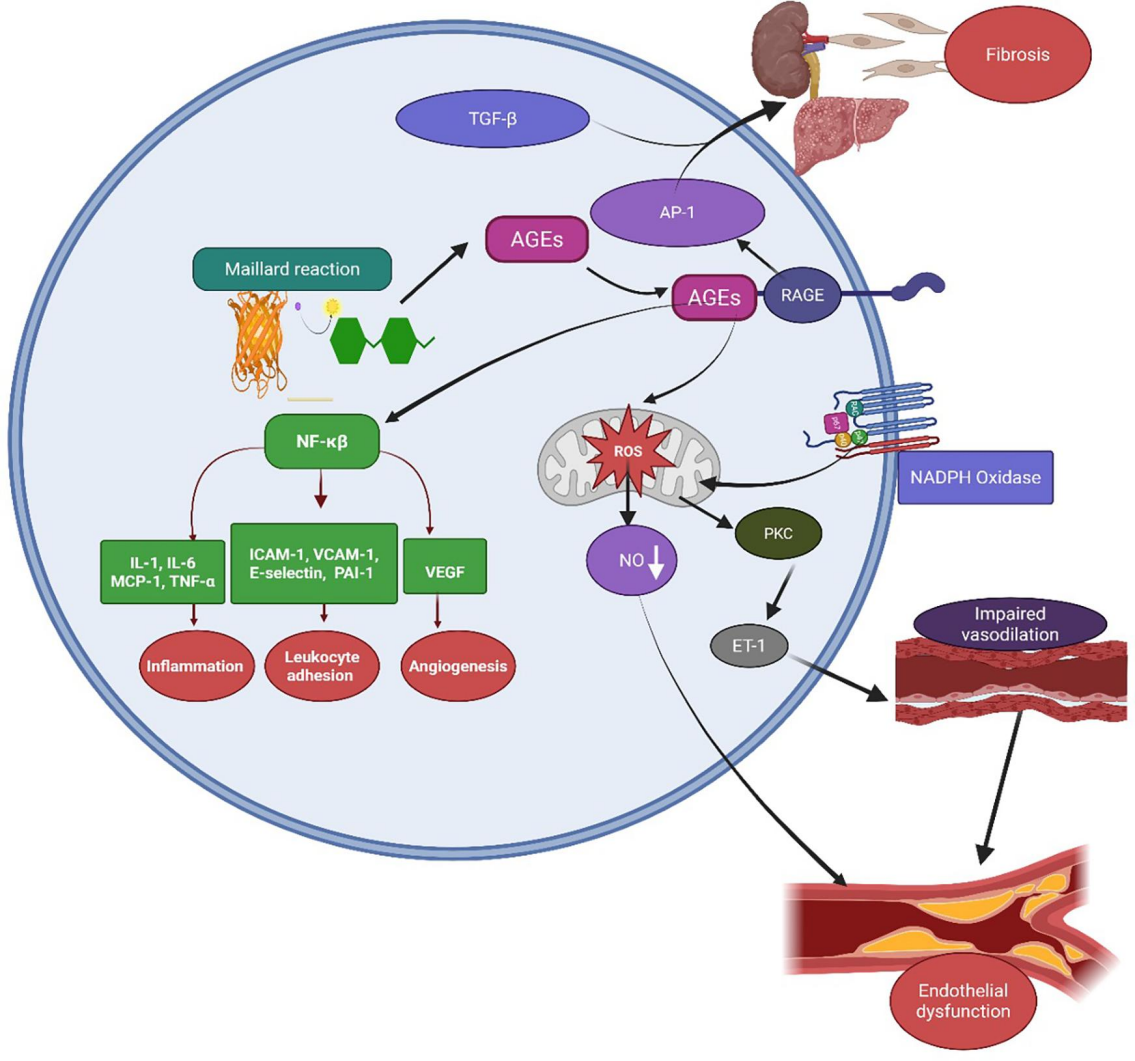
Mechanisms of AGE–RAGE-mediated endothelial dysfunction closing the loop in CRHM syndrome. AGEs, formed through the Maillard reaction under hyperglycemic and oxidative conditions, bind to their RAGE on cell surfaces. This activates intracellular signaling pathways, including NF-κB and AP-1, leading to upregulation of pro-inflammatory cytokines such as IL-1, IL-6, MCP-1, TNF-α, adhesion molecules like ICAM-1, VCAM-1, E-selectin, PAI-1, and VEGF. These mediators promote inflammation, leukocyte adhesion, and angiogenesis. AGE–RAGE interaction also stimulates NADPH oxidase, increasing reactive oxygen species (ROS) production and causing mitochondrial dysfunction. ROS reduce NO bioavailability and activate protein kinase C and endothelin-1 signaling, leading to vasoconstriction, impaired vasodilation, and endothelial dysfunction. Concurrently, TGF-β and AP-1 further increase fibrotic responses in organs such as the kidney and liver, contributing to fibrosis and multi-organ damage. AGEs, advanced glycation end-products; RAGE, receptor for AGE; NF-κB, nuclear factor-kappa B; AP-1, activator protein-1; ROS, reactive oxygen species; NO, nitric oxide; PKC, protein kinase C; ET-1, endothelin-1; TGF-β, transforming growth factor-beta; VEGF—vascular endothelial growth factor; ICAM-1, intercellular adhesion molecule-1; VCAM-1, vascular cell adhesion molecule-1; PAI-1, plasminogen activator inhibitor-1; MCP-1, monocyte chemoattractant protein-1; and TNF-α, tumor necrosis factor-alpha. Created using Biorender, licensed under Academic License.

Persistent hyperglycemia, a hallmark of diabetes and insulin resistance, initiates the Maillard reaction, a non-enzymatic chemical process in which reducing sugars react with amino groups on proteins and lipids, resulting in the formation of AGEs (106). These AGEs accumulate in vascular and parenchymal tissues, where they contribute to arterial stiffness, extracellular matrix remodeling, and atherosclerosis (107, 108). AGEs exert their pathogenic effects primarily through binding to the receptor for RAGE, a transmembrane receptor expressed on endothelial cells, macrophages, and other immune cells, thereby triggering pro-inflammatory and pro-fibrotic signaling cascades (109). Ligand engagement activates intracellular signaling cascades via adaptor proteins such as TIRAP, MyD88, and Dia-1, activating PI3K/AKT, JNK, and MAPKs (p38, ERK). These pathways converge on NF-κB, promoting the transcription of pro-inflammatory cytokines such as IL-6, TNF-α, and MCP-1, while also stimulating NADPH oxidase-dependent ROS production. The resulting oxidative stress amplifies endothelial dysfunction and drives fibrogenesis through AP-1 and TGF-β signaling (110). Furthermore, RAGE activation upregulates adhesion molecules; intercellular adhesion molecule-1 (ICAM-1), vascular cell adhesion molecule-1 (VCAM-1), E-selectin and PAI-1, promoting leukocyte adhesion, thrombosis, and microvascular injury Figure 6 (111). Preclinical studies have shown that sustained AGE exposure induces hallmark features of diabetic kidney disease (DKD) glomerular hypertrophy, fibrosis, and endothelial dysfunction all significantly reduced in RAGE-deficient mice (112). These findings reinforce the central role of the AGE-RAGE axis in the pathogenesis of DKD and broader CRHM syndrome, identifying RAGE as a promising therapeutic target.

Endothelial cells exposed to inflammatory and metabolic stress upregulate cell adhesion molecules including ICAM-1, VCAM-1, and E-selectin. These molecules mediate leukocyte adhesion and transmigration, promoting vascular inflammation and immune cell infiltration into subendothelial tissues (113, 114). Leukocyte-endothelium interactions contribute to atherogenic processes and tissue fibrosis, particularly in the kidneys and liver. This chronic state of endothelial activation establishes a pro-inflammatory vascular niche that augments organ crosstalk and injury, eventually accelerating the progression of CRHM syndrome. Therefore, endothelial dysfunction denotes not only a unifying mechanistic thread but also a critical therapeutic target for breaking the vicious cycle of multisystem failure in CRHM.

### 3.5 Multi-organ damage from inflammation and metabolic dysregulation

#### 3.5.1 Cardiovascular system

Chronic inflammation promotes atherosclerosis and myocardial fibrosis by driving macrophage infiltration and releasing pro-inflammatory cytokines (TNF-α, IL-6, MCP-1), which, in turn, destabilize plaques and contribute to heart failure through TGF-β-driven fibrosis Figure 3 (115). Simultaneously, insulin resistance worsens dyslipidemia and hypertension, altering lipid metabolism and increasing sympathetic nervous system activity, which accelerates coronary artery disease progression (116). Oxidative stress, compounded by mitochondrial dysfunction and NADPH oxidase activation, damages cardiac myocytes, leading to arrhythmias and impaired ventricular function, while the formation of ONOO further disrupts cardiac contractility (117). Meanwhile, endothelial dysfunction compounds these effects by impairing vasodilation, increasing arterial stiffness, and promoting thrombosis, ultimately heightening the risk of hypertension and microvascular ischemia (118).

#### 3.5.2 Renal system (kidneys)

Inflammation, driven TNF-α and IL-6, accelerates glomerulosclerosis and fibrosis, leading to progressive renal dysfunction (119). Insulin resistance and hyperglycemia worsen diabetic nephropathy by causing glomerular hyperfiltration and mesangial expansion, progressing to CKD. Oxidative stress increases ROS production, damaging podocytes and promoting tubular apoptosis, which leads to acute kidney injury and chronic renal dysfunction. Endothelial dysfunction impairs renal microcirculation, reducing glomerular filtration rate (GFR) and promoting renal ischemia, accelerating progression to end-stage renal disease (120). Cardiovascular damage, such as hypertension and atherosclerosis, worsens renal perfusion, while insulin resistance in the liver contributes to renal dysfunction through lipotoxicity (119).

#### 3.5.3 Hepatic system

Inflammation, mediated by TNF-α and IL-6, promotes non-alcoholic steatohepatitis (NASH) through macrophage infiltration and Kupffer cell activation, leading to hepatic fibrosis and cirrhosis. Simultaneously, insulin resistance increases hepatic glucose production and lipogenesis, triggering MASLD and lipotoxicity, which accelerate liver dysfunction (121). Oxidative stress exacerbates this damage by inducing ROS production, lipid peroxidation, and hepatocyte injury, further activating hepatic stellate cells and worsening fibrosis. Endothelial dysfunction impairs hepatic circulation, increasing portal hypertension and leading to complications such as ascites, variceal bleeding, and hepatic insufficiency (122). A recent study found that MASLD, hepatic steatosis, and fibrosis are associated with an increased risk of CKD (123).

#### 3.5.4 Metabolic system

Pro-inflammatory cytokines also impair insulin signaling, leading to hyperglycemia and type 2 diabetes (50). Dyslipidemia-induced lipotoxicity accelerates atherosclerosis, MASLD/NASH, and renal dysfunction (124).

## 4 Novel biomarkers for early detection of CRHM syndrome

Advancements in biomarkers over the past two decades have indeed transformed the understanding and management of cardiovascular, renal, and metabolic disorders (125). Traditional markers like lipid profiles and fasting glucose often miss subclinical organ damage, prompting a shift towards novel biomarkers. Recent studies highlight the significance of biomarkers associated with inflammation, endothelial dysfunction, fibrosis, and metabolic stress. The following biomarkers are crucial for early detection and risk stratification in patients at risk for CRHM syndrome.

### 4.1 Soluble urokinase plasminogen activator receptor

Soluble urokinase plasminogen activator receptor has been extensively studied for its role in chronic inflammation and immune activation. In 2015, a study by Hayek et al. demonstrated that elevated suPAR levels were strongly associated with early kidney dysfunction and endothelial injury, even among individuals with normal renal function (126). A subsequent study by Reiser et al. in 2017 further confirmed that suPAR levels predict glomerular damage and CKD progression, proposing that suPAR interacts with podocyte αvβ3 integrins, thereby promoting glomerular injury (127). These findings position suPAR as an emerging immune-derived biomarker and a potential therapeutic target to mitigate inflammation and cardiovascular risk, particularly in the early stages of CRHM Syndrome Table 1.

**Table 1:** Integrated staging of cardiovascular-renal-hepatic-metabolic syndrome.

Stage	Clinical features	Key biomarkers	Clinical implications
Stage 0 (No Risk Factors)	No metabolic, cardiovascular, hepatic, or renal abnormalities		Encourage lifestyle modifications (healthy diet, physical activity) to prevent metabolic dysfunction
Stage 1 (Dysfunctional Adiposity)	Excess or dysfunctional adiposity	suPAR Galectin-3 GDF-15 miRNAs	Initiate weight management strategies, consider GLP-1RAs (semaglutide) or dual GIP/GLP-1RAs (tirzepatide) for high-risk individuals. Monitor for early metabolic and inflammatory changes
Stage 2 (Metabolic Risk Factors, CKD, or Mild MASLD)	Metabolic risk factors (hypertriglyceridemia, hypertension, MetS, diabetes) or moderate-to-high-risk CKD or MASLD with grade 0/1 fibrosis	NT-proBNP eGFR ACR ALT AST	Initiate statins for ASCVD risk >5%. Control BP <140/90 mmHg in CKD. Consider SGLT2is (dapagliflozin, empagliflozin) for CKD or HF prevention. Screen for MASLD progression
Stage 3 (Subclinical CVD, High-Risk CKD, or MASLD with Fibrosis ≥2)	Subclinical CVD (atherosclerosis, stage B HF) or very-high-risk CKD or MASLD with grade ≥2 fibrosis	suPAR Galectin-3 GDF-15 NT-proBNP ACR	Implement SGLT2is for CKD and HF risk reduction. Consider GLP-1RAs for ASCVD and MASLD. Intensive weight and metabolic control. Early cardiology/nephrology referral
Stage 4a (Without Kidney or Liver Failure)	Clinical ASCVD or HF without kidney/liver failure	NT-proBNP Galectin-3 suPAR GDF-15	Use SGLT2is (dapagliflozin, empagliflozin) and GLP-1RAs (semaglutide) as standard therapy. Dual GIP/GLP-1RAs (tirzepatide) for obesity/metabolic control. Optimize heart failure and ASCVD management
Stage 4b (With Kidney and/or Liver Failure)	Clinical ASCVD or HF with kidney and/or liver failure	eGFR ACR ALT AST NT-proBNP suPAR Galectin-3	Multidisciplinary care approach. Optimize renal and hepatic management. Consider SGLT2is with caution in advanced CKD. Intensify MASLD treatment. Advanced HF therapies if required

ACR, albumin:creatinine ratio; ALT, alanine aminotransferase; AST, aspartate aminotransferase; ASCVD, atherosclerotic cardiovascular disease; CKD, chronic kidney disease; eGFR, estimated glomerular filtration rate; galectin-3, galectin-3; GDF-15, growth differentiation factor-15; GIP/GLP-1RAs, dual glucose-dependent insulinotropic polypeptide/glucagon-like peptide-1 receptor agonists; GLP-1RAs, glucagon-like peptide-1 receptor agonists; HF, heart failure; MASLD, metabolic dysfunction–associated steatotic liver disease; MetS, metabolic syndrome; NT-proBNP, N-terminal pro–B-type natriuretic peptide; SGLT2is, sodium–glucose co-transporter 2 inhibitors; suPAR, soluble urokinase plasminogen activator receptor.

Moreover, suPAR has gained recognition as a robust biomarker of systemic inflammation with significant diagnostic and prognostic utility across diverse clinical settings. Multiple large-scale and cohort studies have demonstrated that elevated suPAR levels predict the onset of CKM syndrome, HF, CKD, and metabolic disorders independently of conventional risk factors and markers like CRP. In a recent UK Biobank cohort study of 25,596 participants, suPAR was a strong independent predictor of CKM outcomes [sub-hazard ratio (sHR)1.23], with a particularly high predictive value for CKD (sHR 1.41) (128). In emergency settings, suPAR exhibited substantial discriminative performance (AUC 0.77) for predicting one-year HF hospitalization and/or mortality, even outperforming NT-proBNP in certain populations (129). Among allogeneic hematopoietic cell transplant recipients, day 7 suPAR levels (AUC 0.75) reliably predicted dialysis-requiring acute kindney injury, outperforming traditional markers such as serum creatinine and neutrophil gelatinase-associated lipocalin (130). In pediatric populations, suPAR levels were significantly elevated in children with obesity compared to those with type 1 diabetes mellitus and healthy controls, reflecting early inflammatory and endothelial disturbances (131). Therefore, these findings underscore suPAR's utility in early disease detection, risk stratification, and outcome prediction across both inpatient and outpatient care. Its ability to reflect immune activation and vascular injury enhances its appeal not only as a diagnostic tool but also as a potential therapeutic target in managing CRHM syndrome and other inflammatory pathologies.

### 4.2 Galectin-3

Galectin-3, a regulatory β-galactoside-binding lectin, plays a central role in chronic inflammation, fibrosis, and tissue remodeling, making it a critical biomarker across a spectrum of cardio-renal-hepatic-metabolic disorders (132, 133). Its clinical significance has been extensively demonstrated in chronic heart failure, particularly heart failure with HFpEF, where elevated serum galectin-3 levels predict new-onset disease, adverse outcomes including all-cause death and cardiovascular death, and correlate with the severity of left ventricular diastolic dysfunction and cardiac structural abnormalities (134, 135). Recent diagnostic studies reveal that galectin-3 has moderate accuracy for identifying HFpEF, with an area under the curve of approximately 0.76, sensitivity of 76%, and specificity of 72% at defined thresholds, outperforming some echocardiographic measures (136). In CKD, galectin-3 serves as a prognostic biomarker linked to increased all-cause mortality and kidney disease progression, with urinary galectin-3 levels correlating with renal fibrosis and decline in eGFR (132, 137). Its expression is closely associated with markers of renal stress and fibrosis, highlighting its potential utility in the early identification of patients at high risk of CKD progression. Although galectin-3's relationship with cardiovascular events in CKD is less consistent, increased levels correlate with endothelial dysfunction and inflammation, suggesting its involvement in vascular pathology within CKD populations (138). Furthermore, galectin-3's association with adverse outcomes is independent of other clinical variables and co-biomarkers such as soluble suppression of tumorigenicity-2, underscoring its unique contribution to disease processes (137). In hepatic disease, particularly in metabolic dysfunction associated fatty liver disease, Galectin-3 has emerged as a promising biomarker for liver fibrosis (139). Its expression correlates with hepatic stellate cell activation, extracellular matrix remodeling, and chronic hepatic inflammation, hallmarks of fibrotic progression (140). Compared to traditional non-invasive fibrosis indices like APRI and FIB-4, Galectin-3 offers superior specificity, especially in early-to-intermediate fibrosis stages. Diagnostic performance improves further when combined with markers such as YKL-40 or imaging techniques like transient elastography (141).

Galectin-3 inhibitors like belapectin are currently under clinical investigation as potential anti-fibrotic therapies in MASLD, offering hope for targeted disease-modifying interventions (142–144). These findings position galectin-3 as a valuable biomarker for risk stratification, diagnosis, and prognosis in patients with overlapping cardio-renal-hepatic-metabolic syndrome. By reflecting underlying inflammatory and fibrotic pathways common to myocardial injury, renal fibrosis, endothelial dysfunction, and metabolic disturbances, galectin-3 integrates these pathologies into a unified concept of multisystem organ dysfunction. This highlights not only its diagnostic and prognostic utility but also its emerging potential as a therapeutic target to interrupt the cycle of chronic inflammation and fibrosis that drives progression in these interconnected diseases.

### 4.3 Growth differentiation factor 15

Growth differentiation factor 15 is emerging as a biomarker involved in inflammation, cellular stress responses, and energy metabolism. Elevated circulating levels of GDF-15 have been shown across various chronic conditions, such as T2DM, CVD, heart failure, obesity, chronic, and CKD, with strong associations with all-cause and cardiovascular mortality (145, 146). Clinically, GDF-15 serves as a non-specific yet prognostically powerful biomarker, particularly in cardiometabolic and inflammatory conditions.

In cardiovascular settings, GDF-15 has demonstrated robust prognostic value. A systematic review of 63 studies confirmed that elevated GDF-15 is an independent predictor of all-cause and cardiovascular mortality, hospitalization, renal dysfunction, and major adverse cardiac events, mainly in patients with HF and atrial fibrillation (145). Recent studies have demonstrated the clinical utility of GDF-15 as a biomarker of disease burden, progression, and prognosis across diverse pathological states. In a prospective study of Fontan patients, elevated baseline serum GDF-15 levels were independently associated with impaired functional status and an increased risk of adverse outcomes, including hospitalization, heart transplant listing, and all-cause mortality. Moreover, patients who exhibited rising GDF-15 levels over a two-year follow-up period were at significantly higher risk of these events, reinforcing the value of serial GDF-15 monitoring (147). Similarly, in patients with MASLD, circulating and hepatic GDF-15 levels were significantly elevated, particularly in those with advanced fibrosis. Notably, treatment with glucagon-like peptide-1 receptor agonists (GLP-1-RA) over two years resulted in a 28% reduction in plasma GDF-15 levels compared to lifestyle advice alone, suggesting a potential role for GDF-15 as a marker of therapeutic response (148).

These findings support the use of GDF-15 as a dynamic, non-invasive biomarker with prognostic and monitoring value across cardiometabolic and hepatic disorders. From a diagnostic perspective, GDF-15's performance in detecting advanced fibrosis in MASLD further supports its clinical applicability beyond traditional liver enzymes, which are often normal in early disease (149). Its non-invasive nature, prognostic utility, and ability to reflect metabolic and inflammatory stress make it a valuable tool in both screening and longitudinal monitoring. Given its cross-organ involvement, GDF-15 can be recommended as a key biomarker in Stage 2 and 3 of CRHM Syndrome Table 1.

### 4.4 N-terminal pro B-type natriuretic peptide

NT-proBNP originally developed as a biomarker for heart failure, has expanded its clinical utility beyond cardiac disease (150). It has shown strong prognostic significance in predicting adverse cardiovascular outcomes, atrial fibrillation, heart failure, and renal complications, particularly in patients with T2DM (151). Recent studies have shown that elevated NT-proBNP (≥125 pg/ml) is associated with increased risks of atrial fibrillation (HR 4.64, 95% CI 2.44–8.85, *p* < 0.001), coronary heart disease (HR 4.21, 95% CI 2.46–7.21, *p* < 0.001), congestive heart failure (HR 4.18, 95% CI 2.18–8.03, *p* < 0.001), and kidney failure (C-index 0.88, 95% CI 0.84–0.92) (152). Similarly, in HFpEF patients, BNP predicted overall adverse events (HR 1.34, 95% CI 1.20–1.52), cardiovascular events (HR 1.36, 95% CI 1.12–1.64), and mortality (HR 1.44, 95% CI 1.04–1.84) (153). NT-proBNP also significantly improved atrial fibrillation risk prediction, with a relative risk of 3.84 (95% CI 3.03–4.87, *p* < 0.001) for highest vs. lowest quartiles (154). Diagnostic assays demonstrated high accuracy, with NT-proBNP rule-out cut-offs (<300 ng/L) showing 96% sensitivity and 95% negative predictive value in acute heart failure diagnosis (AUC = 0.87, *p* < 0.001) (155). The HFriskT2DM-HScore, integrating clinical variables and NT-proBNP, achieved strong discrimination (C-index 0.81, 95% CI 0.80–0.83) and calibration (slope 0.93, 95% CI 0.81–1.1, *p* = 0.31), enabling targeted screening and early intervention in T2DM patients. In a multi-biomarker approach integrating NT-proBNP, creatinine, and albumin has demonstrated strong prognostic utility in valvular heart disease. The resulting cardio-renal-hepatic (CRH) score as an independent predictor of mortality (adjusted HR per 1-point increase: 2.095, 95% CI 1.891–2.320, *p* < 0.001), outperforming conventional risk factors and offering excellent model discrimination (C-index: 0.78–0.81) and net reclassification improvement (0.255, 95% CI 0.204–0.299, *p* < 0.001). These findings underscore the diagnostic accuracy and prognostic relevance of NT-proBNP and BNP in integrated cardio-renal-metabolic assessment, supporting their role in precision medicine approaches for multisystem disease management.

### 4.5 Albumin-creatinine ratio

The albumin-creatinine ratio (ACR) has emerged as a cornerstone biomarker in the integrated staging of cardiovascular renal hepatic metabolic syndrome. Its clinical significance lies in its ability to detect early renal involvement, predict systemic vascular stress, and reflect subclinical target organ damage across multiple systems. CKD, 2DM, hypertension, dyslipidemia, and cardiovascular disease frequently coexist within this syndrome, therefore ACR is a reliable and non-invasive marker to offers substantial clinical utility (156, 157). In a large Chinese cohort study involving 40,188 individuals, elevated ACR levels were strongly associated with increased odds of cardiometabolic conditions. Specifically, individuals in the highest quartile of ACR (≥7.8 mg/g) had significantly higher odds of developing hypertension (OR = 1.56; 95% CI, 1.49–1.64), type 2 diabetes (OR = 1.78; 95% CI, 1.65–1.91), dyslipidemia (OR = 1.39; 95% CI, 1.33–1.46), and cardiovascular disease (OR = 1.12; 95% CI, 1.02–1.23) (158). These associations persisted after adjusting for confounding variables, reinforcing the importance of ACR as a systemic risk indicator, rather than a renal-specific marker alone.

In diabetic care, ACR plays a pivotal role in tracking renal progression. A multicenter Italian study enrolling 1,289 patients with T2DM found that obesity, as measured by increasing waist circumference and body mass index, independently predicted worsening albuminuria over one year. A 5 cm increase in waist circumference conferred a 7% higher risk of albuminuria progression (OR=1.07; 95% CI, 1.00–1.15), while each unit rise in BMI increased the risk by 17% (OR = 1.17; 95% CI, 1.03–1.33) (159). These associations from this study were independent of glycemic control, blood pressure, baseline ACR levels, and therapeutic interventions, highlighting obesity's direct role in renal vascular injury. Therefore, this suggests that routine monitoring of ACR could offer early signals of renal risk escalation in obese diabetic patients, thereby informing timely intervention strategies.

Despite its widespread use, ACR is not without limitations, particularly due to high biological variability. A PREDICT cohort study (*n* = 826) showed a within-individual coefficient of variation of 48.8%, with repeat ACR values ranging from 0.26 to 3.78 times the initial measurement. This degree of fluctuation significantly affects interpretive reliability; a change in urinary ACR from 2 to 5 mg/mmol corresponds to only a 50% probability that a true ≥30% increase has occurred. However, this probability rises sharply to 97% when two spot samples are taken at both time points, underscoring the importance of repeat measurements for accurate monitoring (160). These findings have clear implications for both clinical and research settings, particularly when assessing disease progression or response to therapy.

### 4.6 MicroRNAs

MicroRNAs (miRNAs) play a crucial role in regulating gene expression in cardiovascular and metabolic diseases. In a recent study, miR-126-3p has shown strong prognostic value in predicting long-term cardiovascular events by reflecting endothelial dysfunction, mainly through its association with albuminuria (161). Its elevated levels correlate with an increased risk of coronary artery disease, and hypertension, establishing it as a promising biomarker for cardiovascular risk stratification (162, 163). Similarly, miR-423-5p downregulated in hypertensive patients was shown to mediate protective endothelial effects when overexpressed, pointing to its functional significance in vascular health via the FENDRR/miR-423-5p/Nox4 axis (164). In addition, miR-423-5p is linked to cardiac remodeling and subclinical heart failure supporting its role in Stage 3 and beyond (165). These findings highlight miRNAs as emerging non-invasive biomarkers with potential for early-stage metabolic and cardiovascular risk assessment.

Integrating biomarkers such as eGFR, galectin-3, GDF-15, and NT-proBNP into machine learning models offers a powerful approach to enhance prediction and risk stratification across the cardiovascular renal hepatic metabolic syndrome. These markers reflect distinct yet interconnected pathways of organ dysfunction, and their combined analysis through advanced algorithms can improve early detection and individualized patient management. By leveraging the strengths of these biomarkers within predictive models, clinicians can better identify high-risk patients and tailor interventions, ultimately improving clinical outcomes in this complex multisystem condition.

## 5 Clinical use of SGLT2i, GLP-1ra, and dual agonists in cardiovascular-renal-hepatic-metabolic syndrome

Sodium-glucose cotransporter 2 (SGLT2) inhibitors have redefined management strategies for patients with CKD, heart failure, and type 2 diabetes, all of which are key components of CRHM syndrome. Agents such as dapagliflozin and empagliflozin have demonstrated renal protection by slowing the progression to end-stage kidney disease, independent of glycemic status. Their cardioprotective effects are similarly well established, with significant reductions in hospitalizations for heart failure and cardiovascular mortality, as evidenced in large trials like DAPA-CKD and EMPA-KIDNEY (166, 167). These effects are mediated by hemodynamic and metabolic mechanisms to reduce intraglomerular pressure, promote natriuresis, and attenuate systemic inflammation and oxidative stress. From a biomarker perspective, therapy with SGLT2 inhibitors results in decreased levels of urinary albumin-to-creatinine ratio (UACR), KIM-1, sTNFR1, and NT-proBNP, reflecting improvements in both renal and cardiac stress profiles (168).

### 5.1 GLP-1R agonists

Glucagon-like peptide-1 receptor agonists (GLP-1RAs), such as semaglutide and liraglutide, offer comprehensive metabolic benefits in patients with CRHM syndrome. In addition to reducing hemoglobin A1c and body weight, these agents significantly lower the risk of major adverse cardiovascular events, particularly in patients with established atherosclerotic cardiovascular disease (169). Moreover, GLP-1RAs exhibit anti-inflammatory and hepatoprotective effects, making them suitable for patients with coexisting MASLD. Improvements in liver enzymes such as alanine aminotransferase (ALT) and aspartate aminotransferase (AST) as well as fibrosis markers such as the NAFLD fibrosis score and hepatic steatosis index, have been reported. hsCRP levels also decline with GLP-1RA therapy, supporting their role in systemic inflammation reduction (170).

### 5.2 Dual GIPR/GLP-1r agonists

Tirzepatide, a dual GIP and GLP-1 receptor agonist, is a new therapeutic class with enhanced efficacy in patients with CRHM syndrome. In the SURPASS trials, tirzepatide achieved greater reductions in body weight, glycemia, and lipids compared to GLP-1RAs alone (171). Its effects extend to hepatic improvement, with decreased liver fat content and fibrosis-related markers such as fibroblast growth factor 21 (FGF-21) and procollagen type III (Pro-C3), suggesting a potential to reverse steatosis and early fibrosis. Importantly, tirzepatide also demonstrates reductions in NT-proBNP and UACR, pointing to early benefits in cardiac and renal function, making it an ideal agent for patients with overlapping metabolic, hepatic, and cardiorenal disorders.

### 5.3 Biomarker integration for personalized care

The emergence of biomarker-guided therapy enables precision treatment in CRHM syndrome. Biomarkers such as NT-proBNP, hsCRP, FGF-21, Pro-C3, KIM-1, and suPAR are increasingly used to stratify risk, guide therapeutic decisions, and monitor response. For instance, elevated NT-proBNP may prioritize the use of SGLT2 inhibitors or tirzepatide for patients with cardiac stress, while high hsCRP or Pro-C3 may point to liver inflammation and fibrosis, guiding the use of GLP-1RAs or dual agonists (172). Longitudinal monitoring of these markers can inform adjustments in therapy and predict long-term outcomes, aligning with the goals of organ preservation and metabolic control.

### 5.4 Patient management considerations

In clinical practice, selecting the appropriate pharmacotherapy for patients with CRHM syndrome requires an individualized, patient-centered approach. For patients with diabetic kidney disease or heart failure—particularly those with preserved ejection fraction SGLT2 inhibitors are typically initiated first due to their strong renal and cardiac benefits (21). Regular monitoring of serum creatinine, eGFR, and UACR is necessary to assess treatment efficacy and safety. In patients with obesity, atherosclerosis, or liver dysfunction, GLP-1RAs may be favored, especially when weight loss and improvement in hepatic parameters are treatment goals (3). Liver function tests (ALT, AST), fibrosis indices (NFS), and glycemic markers should be closely monitored. For patients with overlapping metabolic and hepatic abnormalities, especially when rapid weight reduction and enhanced glycemic control are needed, tirzepatide offers a compelling option. Clinicians should also consider patient adherence, route of administration, and comorbidities when choosing therapies.

Combining these agents may also be considered, as their mechanisms are complementary and additive. However, dual-use must be balanced against cost, tolerability, and the need for frequent laboratory monitoring. Overall, the integration of these therapies into routine care alongside lifestyle interventions such as diet, exercise, and smoking cessation marks a comprehensive and progressive approach to managing CRHM syndrome in real-world settings (173).

## 6 Future directions and conclusion

### 6.1 Future directions

The complexity of CRHM syndrome, which arises from the interplay of chronic inflammation, insulin resistance, lipotoxicity, oxidative stress, and neurohormonal dysregulation across the cardiovascular, renal, hepatic, and metabolic systems, calls for innovative, integrated therapeutic strategies. While current therapies such as SGLT2 inhibitors, GLP-1 receptor agonists, and dual GIP/GLP-1 receptor agonists have shown significant organ-protective effects, several gaps in knowledge and practice remain.

Future studies should prioritize the development and validation of integrative biomarkers that reflect the multisystem involvement of CRHM syndrome. These biomarkers can help in early diagnosis, risk stratification, therapy selection, and monitoring of disease progression. Multi-omics technologies—such as genomics, transcriptomics, proteomics, and metabolomics—could uncover novel pathways and identify molecular signatures predictive of treatment response.

Combination therapy trials involving SGLT2 inhibitors and dual incretin agonists should be expanded to evaluate synergistic benefits, especially in diverse populations. There is also a need for long-term cardiovascular, renal, and hepatic outcome data, particularly in patients with overlapping phenotypes like MASLD with CKD or heart failure. In addition, personalized treatment algorithms should be developed, incorporating clinical profiles, biomarker data, and real-world patient-reported outcomes. These would allow stratified treatment approaches that optimize efficacy while minimizing adverse events. Digital health technologies and AI-driven predictive models could further enhance clinical decision-making in CRHM syndrome. Health equity considerations must guide future therapeutic development and implementation. Access to these innovative therapies should be expanded in low- and middle-income countries through policy inclusion, generic drug development, and integration into national chronic disease management programs.

## 7 Conclusion

CRHM syndrome is a progressive, multisystem disorder that requires an integrated approach to diagnosis and treatment. While understanding of its pathophysiology grows, emerging biomarkers like suPAR, Galectin-3, and GDF-15 offer potential for early detection and personalized care but remain underutilized. The rise of SGLT2 inhibitors, GLP-1R agonists, and dual GIPR/GLP-1R agonists marks a therapeutic shift, offering multi-organ benefits. Advancing precision medicine in CRHM will require biomarker integration, individualized therapies, and broader access to innovations. Linking molecular insights with clinical practice is key to improving outcomes and quality of life in affected patients.
